# Distinguishing Between Reservoir Exposure and Human-to-Human Transmission for Emerging Pathogens Using Case Onset Data

**DOI:** 10.1371/currents.outbreaks.e1473d9bfc99d080ca242139a06c455f

**Published:** 2014-03-07

**Authors:** Adam Kucharski, Harriet Mills, Amy Pinsent, Christophe Fraser, Maria Van Kerkhove, Christl A. Donnelly, Steven Riley

**Affiliations:** Department of Infectious Disease Epidemiology, London School of Hygiene & Tropical Medicine, London, UK; Department of Infectious Disease Epidemiology, Imperial College London, London, UK; Department of Infectious Disease Epidemiology, Imperial College London, London, UK; IImperial College London, London, UK; MRC Centre for Outbreak Analysis and Modelling, Imperial College London, London, UK; School of Public Health, Imperial College London, London, UK

**Keywords:** H7N9, infectious diseases, Influenza, statistical inference, zoonoses

## Abstract

Pathogens such as MERS-CoV, influenza A/H5N1 and influenza A/H7N9 are currently generating sporadic clusters of spillover human cases from animal reservoirs. The lack of a clear human epidemic suggests that the basic reproductive number R0 is below or very close to one for all three infections. However, robust cluster-based estimates for low R0 values are still desirable so as to help prioritise scarce resources between different emerging infections and to detect significant changes between clusters and over time. We developed an inferential transmission model capable of distinguishing the signal of human-to-human transmission from the background noise of direct spillover transmission (e.g. from markets or farms). By simulation, we showed that our approach could obtain unbiased estimates of R0, even when the temporal trend in spillover exposure was not fully known, so long as the serial interval of the infection and the timing of a sudden drop in spillover exposure were known (e.g. day of market closure). Applying our method to data from the three largest outbreaks of influenza A/H7N9 outbreak in China in 2013, we found evidence that human-to-human transmission accounted for 13% (95% credible interval 1%–32%) of cases overall. We estimated R0 for the three clusters to be: 0.19 in Shanghai (0.01-0.49), 0.29 in Jiangsu (0.03-0.73); and 0.03 in Zhejiang (0.00-0.22). If a reliable temporal trend for the spillover hazard could be estimated, for example by implementing widespread routine sampling in sentinel markets, it should be possible to estimate sub-critical values of R0 even more accurately. Should a similar strain emerge with R0>1, these methods could give a real-time indication that sustained transmission is occurring with well-characterised uncertainty.

## Introduction

Novel infections that are transmissible between humans but to which there is no immunity have the potential to cause pandemics, sometimes with high morbidity and mortality^1^^,^ 2 ^,^ 3. The majority of emerging infectious disease events are caused by zoonotic pathogens, most of which have their origins in wildlife, as SARS or avian influenza do^4^. The frequency of zoonotic (or spillover) events, where pathogens are transmitted to novel hosts from reservoir species, has increased in recent decades and such events pose a substantial risk to human populations^5^.

Various factors need to be considered when assessing a new threat from disease lineages that circulate in animal populations, including: their rate of infection in domestic animals; the frequency with which they infect humans; the severity of infection in humans; levels of pre-existing immunity in the human population; and the rate at which they are adapting to human hosts^6^^,^ 7. However, the current capability of a pathogen to transmit from human to human is of paramount importance^8^^,^ 9. If the basic reproductive number, R_0_, defined as the average number of secondary cases generated by a typical infectious case in a fully susceptible population, is less than one, the virus will not cause a pandemic^10^. The closer R_0 _is to one, the lower the hurdle that must be overcome for the strain to persist.

There are two central objectives for the surveillance of novel human infections: first, to quickly detect zoonotic events and assess their spillover threat; and second, to rapidly and consistently detect temporal changes in the degree of transmissibility between humans. To disentangle the role of animal-to-human and human-to-human transmission, we present a model of spillover exposure and onwards human-to-human transmission in which human cases on a given day can arise from exposure to animals or as a result of earlier human cases. We do not assume that the temporal pattern of exposure to animals (the spillover hazard) is known and we jointly estimate spillover exposure and the human-to-human R_0_. Using simulated data, we first examine the feasibility of obtaining robust estimates of R_0 _from reported (human) cases. We then apply the method to real case data, estimating the value of R_0 _in the three largest outbreaks reported for influenza A/H7N9 from China: Shanghai (33 cases), Zhejiang (46 cases) and Jiangsu (27 cases).

## Methods

**Epidemic model**

In our transmission model, human cases could be generated in one of two ways. First, they could arise from exposure to animals. We defined *h_A_(t)* to be the expected number of new human cases with onset on day t due to exposure to animals. This was assumed to be a step function with S steps and *S − 1* change points. Cases could also arise from human-to-human transmission: we assumed infected individuals had an infectiousness profile described by a Poisson distribution with mean *λ*, the serial interval of the disease. The number of new infections generated by each infectious individual depended on R_0 _and, because there were few total infections relative to the population size, we assumed no saturation effects: depletion of the susceptible pool did not affect the dynamics. Our baseline assumption was that the offspring distribution followed a Poisson distribution. We defined *h_H_(t)* to be the expected number of new human cases with onset on day t due to previous human cases,

\begin{equation*}h_H(t) = \sum_{i=1}^{I_t} R_0 \frac{ \lambda^{(t-d_i)} e^{-\lambda}}{(t-d_i)!} ~, \end{equation*}

where *d*_i_ was the day infected (so *t − d_i_* was the days since individual *i* was infected) and *I_t_* was the total number of infected individuals at time t. The number of new human cases each day, *N*_t_, was also chosen from a Poisson distribution with mean *h_A_(t) + h_H_(t)*.

**Statistical inference**

Given a parameter set *θ*, the likelihood of a time series of observed human cases *{N_1_,…,N_T_}* was^9^:

\begin{equation*}L( \theta | N)  = \prod_{t=0}^{T-1}   \frac{\mu_t^{N_{t+1}} e^{-\mu_t}}{N_{t+1}!}\end{equation*}

where

\begin{equation*}\mu_t= \left\{\%0A\begin{array}{c c}\%0Ah_A(t,\theta)  \%26 \text{if } t= 0~ ; \\\%0A\sum_{i=1}^{\min(k,t)} R_0 N_{t-i+1} \frac{\lambda^i e^{-\lambda}}{i!}+h_A(t,\theta) \%26 \text{if } t>0\%0A\end{array} \right.\end{equation*}

and *k* is the maximum value the serial interval distribution can take. Model inference was performed using the full likelihood and Markov Chain Monte Carlo (MCMC) over the space of possible parameter values. Each parameter was assumed to be positive, with a flat linear prior distribution otherwise.

As a sensitivity analysis, we also considered the possibility of data arising from an overdispersed offspring distribution while inference was performed assuming a Poisson distribution. For this analysis, secondary cases were drawn from a negative binomial distribution with mean R_0_ and shape parameter 0.1.

**H7N9 case data**

Between 19th February and 10th August 2013, there were 136 reported human cases of influenza A/H7N9 in China (including one asymptomatic). We considered the three provinces with the largest number of cases: Shanghai, Zhejiang and Jiangsu. The first H7N9 case was recorded in Shanghai on 19th February. As a result of the outbreak, all live bird markets (LBMs) in Shanghai were closed on 6th April. The last recorded onset date in Shanghai in the 2013 outbreak was 13th April, and the total number of cases was 33 (with onset dates known for 29). The first case in Zhejiang had onset date 7th March and the last had onset date 18th April; the total number of cases was 46 (all with known onset date). Of the 35 cases with known location, 23 occurred in Hangzhou, the capital of Zhejiang and 9 in Huzhou, a prefecture-level city just north of Hangzhou. In Hangzhou LBMs were closed on April 15th and in Huzhou LBMs were closed around April 10th. In Jiangsu, the first case had onset date 8th March and the last 19th April; in total there were 27 cases, 23 with known onset date. Twenty cases have known location, half of which were in Nanjing, the province capital, where LBMs were closed on April 6th. We gathered data from a variety of public sources including: ProMed, WHO, FluTrackers, news reports and research articles^11^. The line list is available on Dryad (doi:10.5061/dryad.2g43n).

## Results

**Estimating basic reproduction number and spillover hazard**

Testing our novel statistical framework against simulated case data, we found that the model could distinguish between human-to-human and animal-to-human transmission when the basic reproductive number was substanitally greater than zero (R_0 _= 0.6), but still subcritical.

**Simulation results with mean serial interval λ=6 days and R0=0.6.**
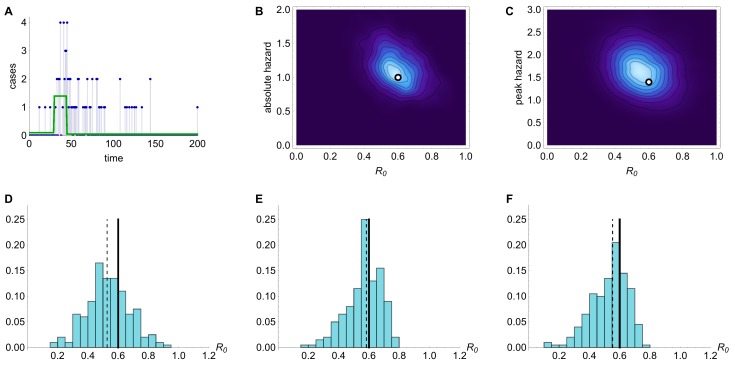
Results from inference using simulated time series with R_0 _=0.6 and mean serial interval λ=6. (A) Time series generated by model, with cases as blue points and spillover hazard function given by green line. (B) Joint posterior distribution for R_0 _and absolute spillover hazard from model inference when timing and relative amplitude of spillover hazard are known. True values are indicated by white dot. (C) Joint posterior distribution for R_0 _and peak spillover hazard (i.e. middle step of hazard function) when amplitude and timings of the spillover hazard steps are unknown but the hazard drop date (e.g. date of market closure) is known. (D) Histogram of estimated value of R_0 _from model inference using 200 different simulated time series, when neither amplitude nor timings of the spillover hazard steps are known. Each value is calculated as the median of the posterior distribution for R_0_. Solid lines, R_0 _in the simulated data. Dashed lines, the inferred value of R_0_. (E) Histogram of estimated value of R_0_ when relative amplitude and timings of the spillover hazard steps are known. (F) Histogram of estimated value of R_0 _when amplitude and timings of the spillover hazard steps are unknown but the hazard drop date is known.

We simulated 200 different time series with R_0_ of 0.6 and a three step spillover hazard (Fig. 1A). We assumed that λ, the serial interval for infections (the average time from onset of a primary case to onset of a resulting secondary case), was 6 days (results for λ=3 are shown in Figure 2). Ideally, we would be able to measure – via virological market surveillance or otherwise – the change in the relative spillover hazard over time, leaving just two unknowns: R_0 _and the absolute magnitude of the spillover hazard. Fig. 1B shows that if we assumed the amplitudes and timings of the steps in the spillover hazard were known, our estimates of R_0 _were tightly constrained around the true value (Fig. 1E). With current surveillance practices it is likely that far less about the spillover hazard will be known. However, if the risk of transmission from animals is decreased by culling or market closure, this date is often reported^12^. Therefore we modelled a known drop in spillover hazard date (±7 days), but with nothing else known about the shape or magnitude of the hazard (Fig. 1C). Even in this scenario, we obtained unbiased estimates of R_0_, although the posterior distributions had higher variance than before (Fig. 1F). In contrast, if we had no information whatsoever about the shape and magnitude of the spillover hazard, we obtained a diffuse posterior distribution, with an apparently biased median value for R_0_ (Fig. 1D).

**Simulation results when mean serial interval λ=3.**
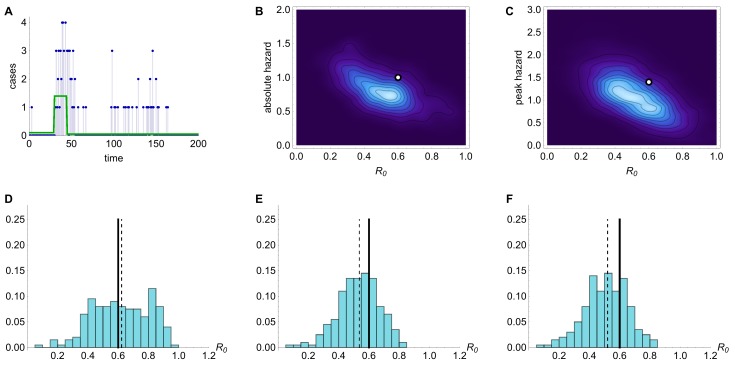
Results from inference using simulated time series with R_0 _=0.6 and mean serial interval λ=3. (A) Time series generated by model. (B) Joint posterior distribution for R_0 _and absolute spillover hazard from model inference when timing and relative amplitude of spillover hazard are known. (C) Joint posterior distribution for R_0 _and peak spillover hazard when amplitude and timings of the spillover hazard steps are unknown but the hazard drop date is known. (D) Histogram of estimated value of R_0 _from model inference using 200 different simulated time series, when neither amplitude nor timings of the spillover hazard steps are known. (E) Histogram of estimated value of R_0 _when relative amplitude and timings of the spillover hazard steps are known. (F) Histogram of estimated value of R_0 _when amplitude and timings of the spillover hazard steps are unknown but the hazard drop date is known.

When secondary cases are generated using a negative binomial distribution with mean equal to R_0_, and overdispersion parameter 0.1, but inference is performed assuming a Poisson offspring distribution, our estimates for R_0_ are below the true value (Fig. 3). However, even when the offspring distribution is mis-specified in this way, it is still possible to detect a signature of human-to-human transmission from onset data.

**Sensitivity to mis-specification of offspring distribution:**
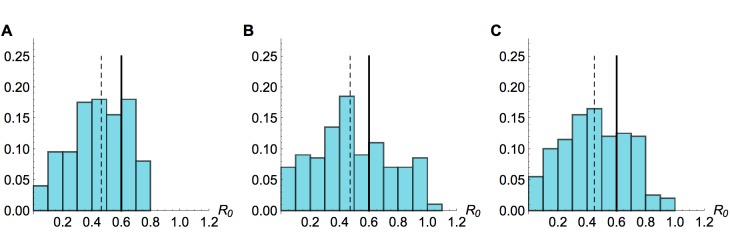
Results from inference using simulated time series with R_0_=0.6 and λ=6, with a negative binomial offspring distribution in simulations, and Poisson distribution in the inference model. (A) Histogram of estimated value of R0 from model inference using 200 different simulated time series, when neither amplitude nor timings of the spillover hazard steps are known. (B) Histogram of estimated value of R0 when relative amplitude and timings of the spillover hazard steps are known. (C) Histogram of estimated value of R0 when amplitude and timings of the spillover hazard steps are unknown but the hazard drop date is known.

Our model also appears to be insensitive to functional mis-specification of the spillover hazard function, so long as the timing of the drop in hazard is known. In order to examine the sensitivity of our estimates to a mis-specified hazard function, we generated 200 time series using an exponential growth of hazard from spillover exposure and fitted a step-wise spillover hazard function in our analysis (Fig. 4A). Taking the median estimate of R_0 _from the posterior distributions of the model fits, we found that even with this mis-specification we obtained an unbiased estimate of R_0_ (Figs. 4B–C).

**Sensitivity to mis-specification of hazard function:**
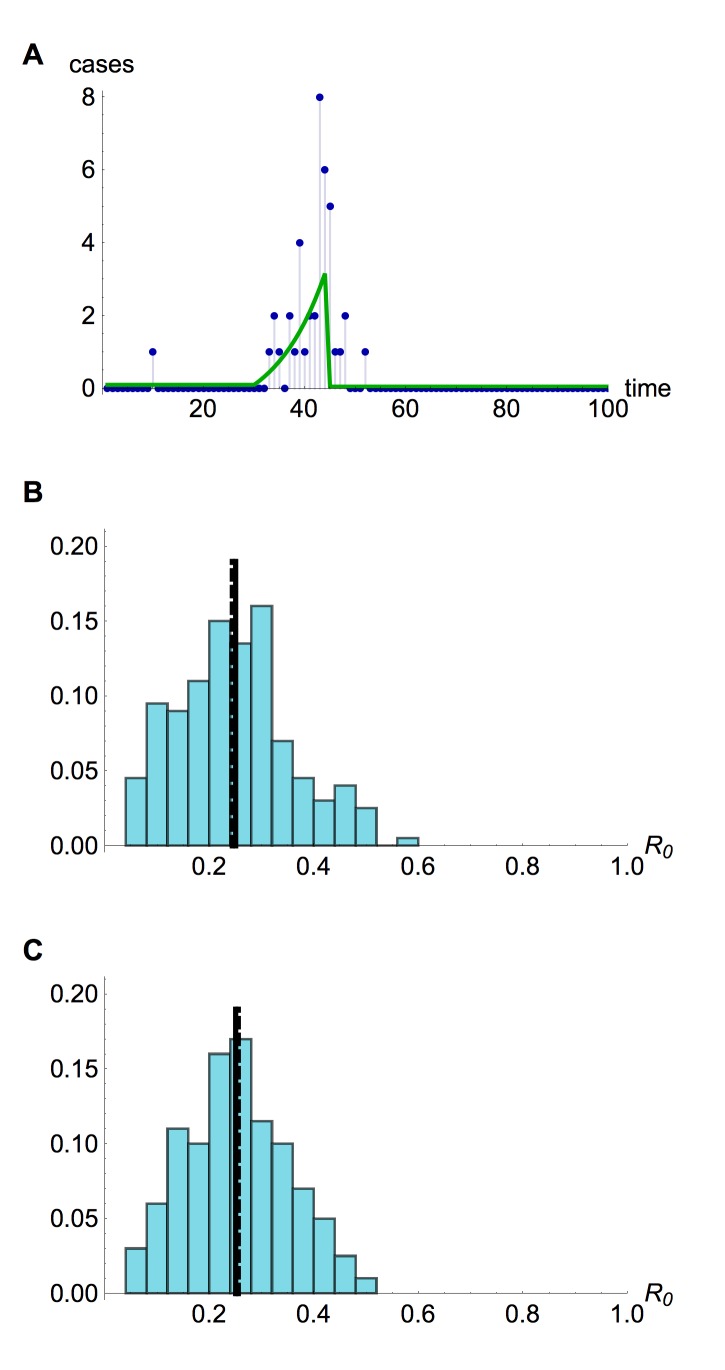
Model inference when simulated spillover hazard depends on an exponential function, and inference model uses a step function. (A) Representative run when R0 =0.25 and λ=3. Blue points, simulated time series; green line, hazard function in the case generation model. (B) Histogram of median posterior distribution for R0 for 200 time series generated with λ=3. Black line shows true value of R0 =0.25; dashed line, median of inferred values. (C) Histogram of median posterior distribution for R0 for 200 such time series with λ=6.

To test how well the model estimated the proportion of cases that arose from human-human transmission, we simulated 200 time series and recorded whether each case came from an animal-human or human-human source. Fig. 5A shows that the model generally provides good estimates of the proportion of human-human cases, and generates underestimates more often than overestimates. Fig. 5B shows that when simulated secondary cases are overdispersed, there is more variation in estimates of the proportion of human-human transmission. However, the inferred and true values are still strongly correlated: in both cases the Pearson correlation coefficient is >0.8 (p<0.0001).

**Inference of proportion of human-human transmission:**
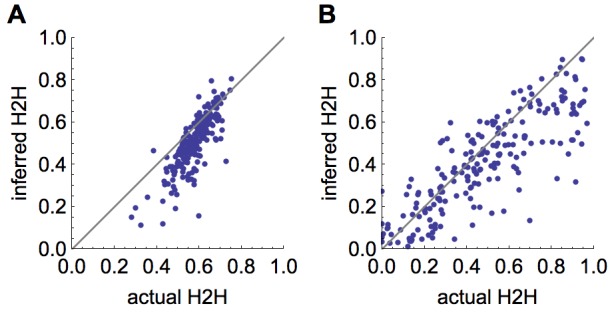
(A) Comparison of inferred proportion of cases resulting from human-human transmission and actual proportion, for 200 simulated timeseries, using a Poisson offspring distribution. (B) Comparison of inferred human-human cases and actual proportion when offspring distribution is negative binomial with shape parameter 0.1.

**Influenza A/H7N9 in China**

Applying our methods to data from the recent outbreak of influenza A/H7N9 in China, with spillover resulting from exposure to poultry in live bird markets, we found support for the presence of human-to-human transmission, but no evidence that R_0 _was near the critical value of one. First we calibrated the animal exposure portion of the model in the absence of human-to-human transmission, setting R_0_=0 and estimating a stepwise hazard with arbitrarily many steps. This framework contains the saturated likelihood model: with a step for every day, we obtain the highest possible probability for the data. However, only a few steps were required for a parsimonious model of animal-to-human-only infections: using the Bayesian Information Criterion (BIC), we see that the models with three or four steps have substantially more support than other hazard functions across Shanghai, Zhejiang and Jiangsu (Table 1). Therefore we used a three step function in the rest of our analysis. Next, we added human-to-human transmission to the spillover model with a three step hazard function and known date for a decrease in hazard (in this instance, the market closure date). Based on cluster data available at the time of the outbreak^13^, we first assumed λ=9.6 days. Table 1 shows that the model with human-to-human transmission had more support than the animal-to-human-only model for Shanghai and Jiangsu, with both models having similar support for Zhejiang.

**Table 1: Comparison of different models of spillover hazard.** 

Region	Model	Likelihood	Parameters	BIC
Shanghai	2 step	-51.7	3	115.8
	3 step	-40.7	5	102.3
	4 step	-39.8	7	108.6
	5 step	-37.5	9	112.5
	6 step	-39.5	11	124.8
	7 step	-37.5	13	129.1
	Exponential	-41.4	5	103.5
	Log-normal	-38.9	7	106.9
	Gamma	-39	7	107.1
	3 step with H2H	-38	6	100.9
Jiangsu	2 step	-39.6	3	91.8
	3 step	-36.4	5	93.7
	4 step	-35.3	7	99.8
	5 step	-36.1	9	109.9
	6 step	-36.4	11	118.9
	7 step	-36.9	13	128.3
	Exponential	-39.7	5	100.3
	Log-normal	-35.8	7	101
	Gamma	-36.8	7	103
	3 step with H2H	-33	6	91.1
Zhejiang	2 step	-82.8	3	178.2
	3 step	-48.1	5	117.2
	4 step	-44.4	7	118.3
	5 step	-43.5	9	124.9
	6 step	-46.1	11	138.4
	7 step	-44.6	13	143.9
	Exponential	-48.9	5	118.9
	Log-normal	-47.9	7	125.2
	Gamma	-48	7	125.5
	3 step with H2H	-46.9	6	119.1

In all three datasets, the estimate of R_0 _is well below one and the 95% credible interval around the estimate always excluded one (Table 2). There was evidence of heterogeneity in R_0 _between these three outbreaks, with two having very similar posterior densities: Shanghai – median 0.19 (95% credible interval 0.01–0.49), and Jiangsu 0.29 (0.03-0.73); and one with support only for much lower values of R_0_: Zhejiang 0.03 (0.00–0.22).

**Table 2: Estimate of R0 in different regions. 95% credible interval is given in parentheses.** 

Region	λ	R_0_ estimate
Shanghai	3	0.39 (0.02-0.90)
	6	0.29 (0.04-0.66)
	9.6	0.19 (0.01-0.49)
Jiangsu	3	0.32 (0.02-0.78)
	6	0.22 (0.01-0.58)
	9.6	0.29 (0.03-0.73)
Zhejiang	3	0.26 (0.00-0.69)
	6	0.11 (0.00-0.37)
	9.6	0.03 (0.00-0.22)

Figures 6A–C show the posterior distributions for R_0_, the timing of all other changes in the spillover hazard and the amplitude of the spillover hazard. Despite using only case onset data from publicly available sources, these values are consistent with recently published estimates for R_0_ based on non-public data obtained during subsequent investigations by China CDC^14^, which were not available at the time of the outbreak.

**Estimates for R0 and spillover hazard for A/H7N9 in China:**
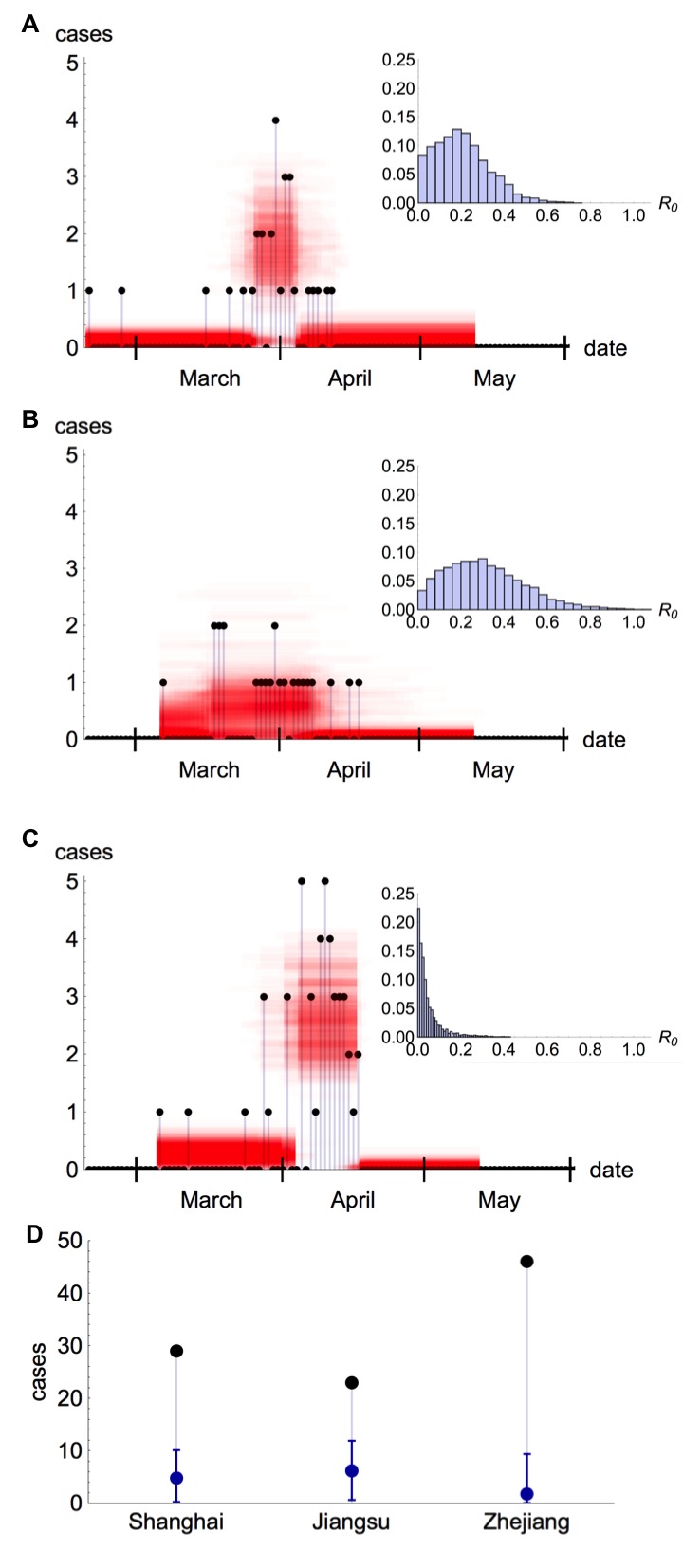
Posterior estimates for spillover hazard (in this case resulting from live bird markets) and R_0 _for influenza A/H7N9 in China, assuming a mean serial interval λ=9.6. (A) Case incidence for Shanghai and the spillover hazard from the best fitting model with market and human-to-human hazard: black dots, observed H7N9 cases; red shaded region, posterior distribution for amplitude of the spillover hazard. Inset: Posterior distribution for R_0 _in Shanghai. (B) Case incidence for Jiangsu and inferred spillover hazard in best fitting model. (C) Case incidence for Zhejiang and inferred spillover hazard in best fitting model. (D) Estimated number of cases resulting from human-to-human transmission in different regions. Black points, total observed onsets; blue points, estimated non-index cases (with error bars representing 95% credible interval).

We were also able to estimate the number of observed non-index human cases that resulted from human-to-human transmission (Table 3). Overall, for these three outbreaks, we estimated that 13% (1%–32%) of observed cases arose from human-to-human transmission. The relative patterns in our estimates for R_0 _and our finding that R_0 _is below one were unaffected if we instead assumed a smaller serial interval^15^^,^ 16, one of 6 days rather than 9.6 days (Table 2). However, a serial interval of 3 days does increase the estimate of R_0 _in Zhejiang.

**Table 3: Estimated number of cases resulting from human-to-human transmission in different regions.** 

Region	Observed onsets	λ	Non-index cases (95% credible interval)
Shanghai	29	3	8.5 (1.0-22.0)
		6	5.9 (0.6-10.5)
		9.6	6.9 (0.9-10.7)
Jiangsu	23	3	8.2 (0.9-14.9)
		6	5.7 (0.38 -14.6)
		9.6	4.4 (1.1-11.8)
Zhejiang	46	3	10.6 (1.4-25.5)
		6	3.0 (0.13-22.0)
		9.6	1.5 (0.1-6.5)

There has been some speculation that not all cases of influenza A/H7N9 were reported^17^, however our method produced reliable estimates for R_0 _even if simulated time series were subject to partial reporting. We considered time series with a mean of 33 observed cases (the average number of observed cases with known onset across the three regions of China we examined), with only 1% or 25% of actual cases observed (Fig. 7).

**Sensitivity to under-reporting when drop in hazard known:**
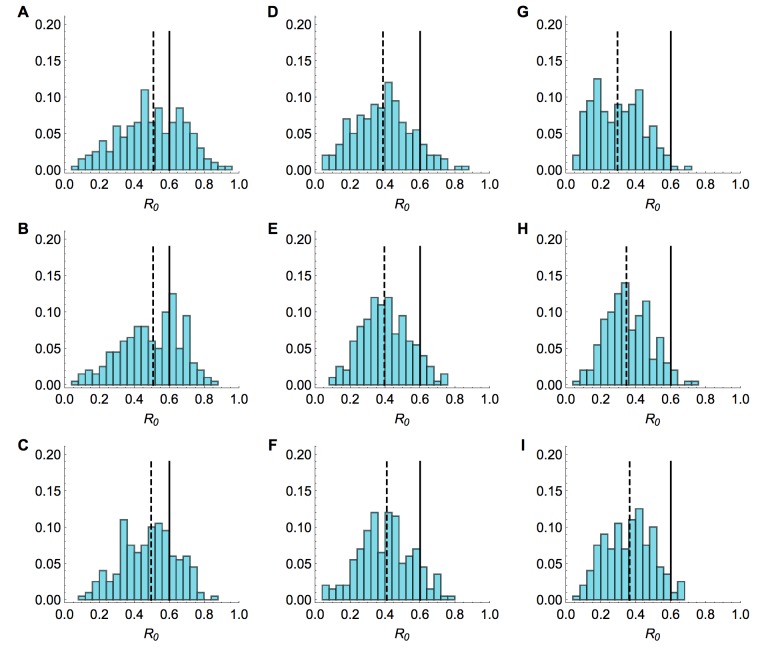
Histograms of estimated value of R0 from model inference with simulated time series when only timing of drop in spillover hazard known. Market parameters are chosen so that a mean of 33 cases are observed, regardless of reporting rate. 200 different time series were simulated. Each value in the histogram is calculated as the median of the posterior distribution for R0. Solid lines, R_0_ = 0.6 in the simulated data; dashed line, median of inferred values. Top row, serial interval is 3 days; middle row, 6 days; bottom row, 9.6 days. (A), (B) and (C) All cases observed. (D), (E) and (F) only 25% of cases observed. (G), (H) and (I) Only 1% of cases observed.

As was the case with full reporting, the R_0 _estimates were improved when more information about the hazard function was known (Fig. 8). Although underreporting is a common problem in outbreaks for multiple reasons, these results suggest that our framework could be used reliably even when the true extent of the outbreak is unknown, assuming that cases are reported with a constant probability over time.

**Sensitivity to under-reporting when shape of hazard known:**
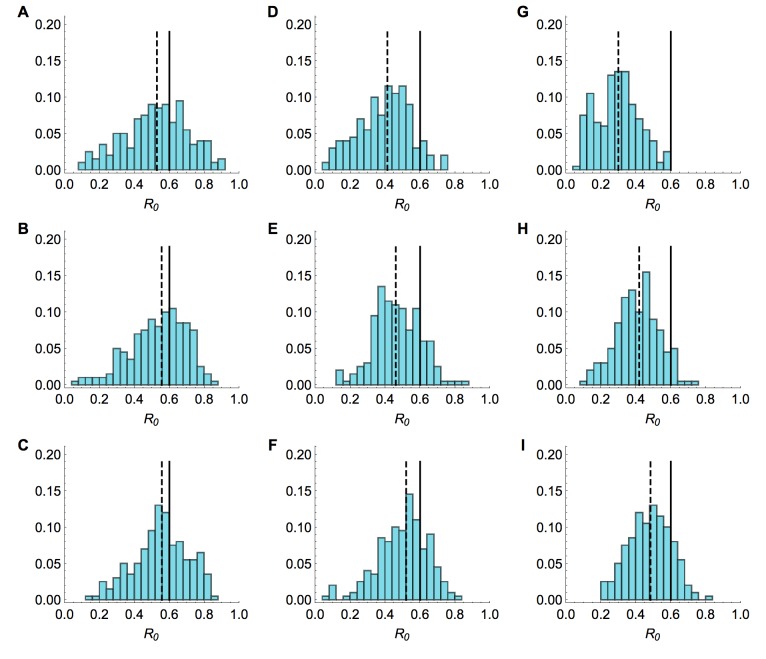
Histograms of estimated value of R0 from model inference simulated time series when relative amplitude and timings of spillover hazard known. Market parameters are chosen so that a mean of 33 cases are observed, regardless of reporting rate. 200 different time series were simulated. Each value in the histogram is calculated as the median of the posterior distribution for R0. Solid lines, R0 = 0.6 in the simulated data. Top row, serial interval is 3 days; middle row, 6 days; bottom row, 9.6 days. (A), (B) and (C) All cases observed. (D), (E) and (F) only 25% of cases observed. (G), (H) and (I) Only 1% of cases observed.

**Emergence of a human transmissible strain**

Should similar outbreaks occur in future with R_0 _> 1, these methods could be valuable in real time to generate the earliest possible evidence of sustained human-to-human transmission. Using a simulated time series with R_0 _= 1.05 (Fig. 9A), and assuming only the timing of drop in spillover hazard is known, we found our estimate of R_0 _approached the true value once the spillover hazard decreased, although the credible interval still included many values below one (Fig. 9B). In contrast, if the shape of the spillover hazard – but not the overall amplitude – was known, we obtained useful information about R_0_, and hence the presence of sustained human-to-human transmission, much earlier (Fig. 9C).

**Estimation of R0 in real time:**
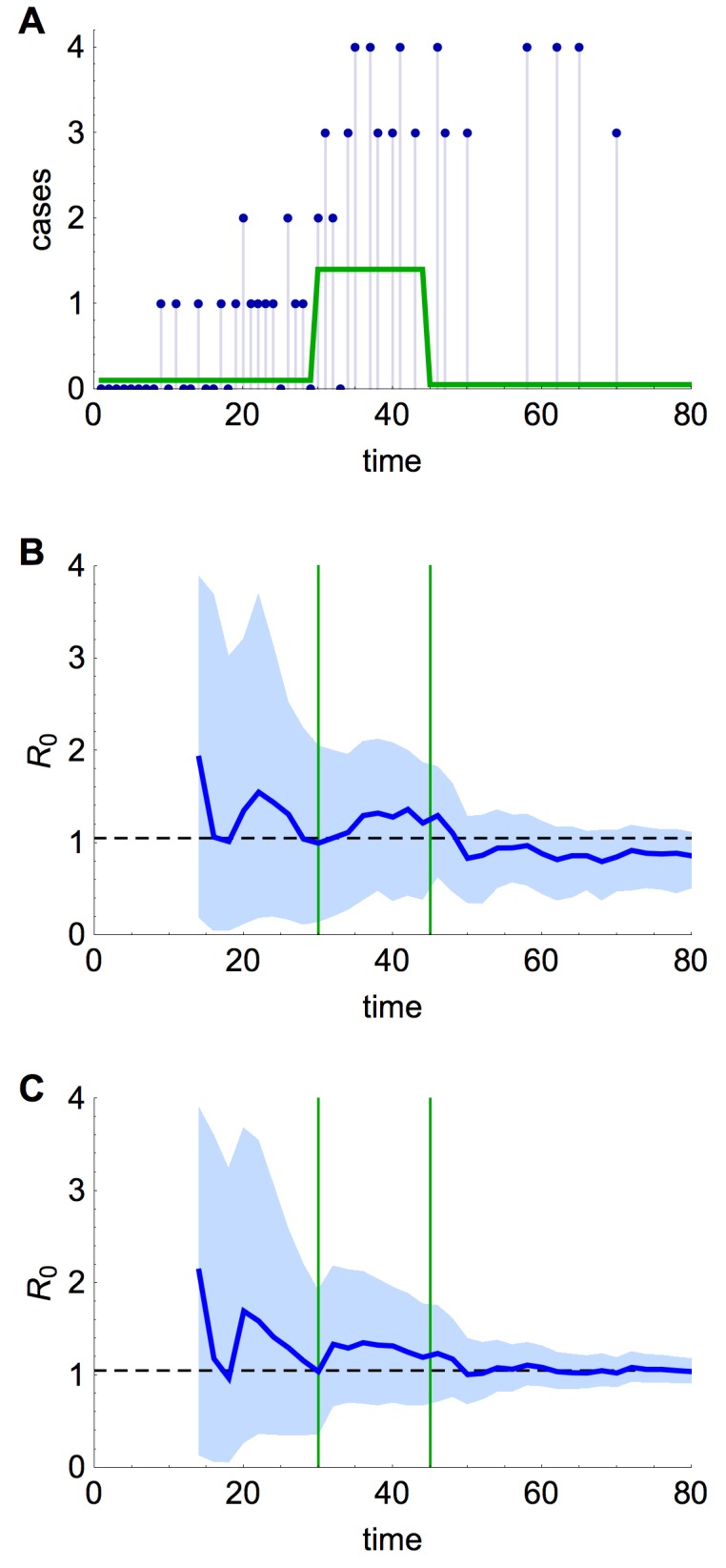
(A) Simulated time series, with cases as blue points and spillover hazard function given by green line. R_0 _= 1.05 and λ = 6. (B) Real time estimate of R_0 _when only the timing of drop in spillover hazard was known. Thick blue line, inferred estimate of R_0 _at that point in time; blue shaded region, 95% credible interval; dashed line, true value of R_0_. (C) Real time estimate of R_0 _when shape of the spillover hazard but not the overall amplitude was known.

## Discussion

The statistical framework presented here would have substantial value during future outbreaks so long as the timing of drops in hazard are known. Also, if direct evidence of the parametric form of the spillover hazard function over time could be obtained, these methods would provide even more accurate results. With a sufficiently long case time series, repeated simulation and parameter fitting suggests that our model produces unbiased estimates of the basic reproductive number, R_0_. This is the case even when there is underreporting of cases and when the spillover hazard function is mis-specified as a step function when simulated with exponential growth. We were also able to recover parameters when data were simulated using an overdispersed offspring distribution, and inferred under the assumption of Poisson distributed secondary cases.

Our method adds to the currently available statistical toolkit for analyzing spillover infections. Previous studies have obtained estimates for R_0 _using data about household-based cases (influenza A/H5N1^18^ and A/H7N7^19^), or by utilizing knowledge that some cases were of animal origin and others were not (influenza A/H3N2v^20^, influenza A/H5N1^21^ and monkeypox^22^). Using our model, it is possible to detect a signature of human-to-human transmission for a spillover infection from only the time series of overall clinical incidence.

There are some limitations to the framework and results we have presented. Firstly, we were not able to jointly estimate either the mean or variance of the serial interval distribution for human-to-human infections. Rather, we assumed a value using the best currently available evidence and then tested the sensitivity of key results to changes in that value. Also, we used only publicly available data; additional evidence has become available since 2013 and has confirmed our estimates^14^. Our reliance on only onset data and not outbreak investigation data can be viewed as a strength: case counts by day of onset are often the earliest data public health decision makers in both local and remote populations have access to when making rapid assessments of risk during a spillover event. Despite these limitations, our formulation adds substantial additional insight to the time series of new cases.

We found evidence that around 13% of observed cases in the three largest clusters of influenza A/H7N9 in China in 2013 resulted from human-to-human transmission, rather than from spillover exposure (this increased to 15% when we assumed a serial interval of 6 rather than 9.6 days). This contrasts with the five potential cases of human-to human-transmission reported out of a total of 136 cases^23^^,^ 24, suggesting that a greater number of human-to-human cases may have been confirmed if additional data on each case’s potential for exposure had been available. It is interesting that the peak of our posterior estimates for R_0 _in Shanghai and Jiangsu (Figs. 6A–B) is above zero, but for the slightly later epidemic in Zhejiang there is no evidence that R_0 _is significantly greater than zero (Fig. 6C). These differences in R_0 _posteriors could be explained by a number of hypotheses. It could be that changes in behaviour – perhaps as a reaction to mass media reports – reduced transmission by the time the Zhejiang outbreak started. Alternatively, differences in human population structure and density, as well as the distribution of markets and bird movements, could have affected the dynamics of the infection in different regions.

The framework we present has substantial potential value for public health. First, the approach can be applied to any outbreak of a spillover infection similar to influenza A, and gives a useful upper bound for R_0 _consistent with the observed onset data. Second, the results illustrate how being able to characterise the variation in spillover hazard over time can permit accurate estimates of R_0 _from relatively small outbreaks. For example, to characterise the hazard from avian influenza in poultry markets, weekly random samples could be taken and stored from sentinel markets. In the event of a nearby outbreak of human cases, stored samples could be tested and the temporal variation in spillover risk estimated independent of the incidence of human cases. If this approach were combined with routine sequencing of human isolates, it is possible that fitness increases in the virus could be observed prior to the virus crossing the critical threshold of R_0 _equal to one.

## Author contributions

AJK, HLM and SR designed the study and wrote the paper. AJK and HLM performed the study. AJK, HLM, AP, CF, MDVK, CAD and SR analyzed the data. AJK and HLM contributed equally to the work.

## Competing Interests

The authors have declared that no competing interests exist.
